# Quadratic decomposition based TCN-Transformer for time series prediction of micro-seismic signals in coal mines

**DOI:** 10.1038/s41598-025-22832-3

**Published:** 2025-11-10

**Authors:** Yuanping Gan, Chao Huang, Zuoli Zhang, Weidong Lu, Bingpeng Gao, Xin Cai, Tao Xu

**Affiliations:** 1 School of Safety Science and Engineering, Xinjiang College of Engineering, Urumqi, 830000 China; 2 Xinjiang Key Laboratory of Intelligent Prevention, Control and Emergency Response to Coal Mine Disasters, Urumqi, 830000 China; 3 School of Intelligence Science and Technology, Xinjiang University, Urumqi, 830017 China

**Keywords:** Micro-seismic signals, Time series prediction, Quadratic mode decomposition, Fuzzy entropy, Deep learning, Engineering, Information technology

## Abstract

As shallow coal reserves are diminishing in China, mining operations are extended to deeper levels, such that characteristics like high geopressure, intense gas adsorption, and reduced permeability become obvious. The mining environment alters significantly. To monitor geological hazards including rock burst during coal mining, this paper presents a time series prediction model for micro-seismic signals by quadratic modal decomposition and a TCN-Transformer network. At first, the micro-seismic signal is primarily decomposed by CEEMDAN. The decomposed Intrinsic Mode Functions (IMFs) are classified and reconstructed by fuzzy entropy. Then, a secondary decomposition is performed by VMD to uncover the signal’s latent features. Thereafter, the time series prediction model is developed by integrating the TCN network’s multi-scale feature extraction capabilities with the self-attention mechanism of the Transformer network. The experimental results demonstrate that the model effectively captures both local and global features within micro-seismic signals for enhancing prediction accuracy. Validation with micro-seismic monitoring data from an actual coal mine in Xinjiang confirms the model’s strong fitting ability and robustness, and further indicates the early warning capabilities for rock robust. The proposed method can offer reliable technical support for safe coal mine operations.

## Introduction

As a major energy consumer, China has a natural energy structure characterized by an abundance of coal, scarcity of oil, and limited gas, which indicates that coal will remain the primary energy source for an extended period in the future^1,2^. As the depletion of China’s shallow coal resources intensifies, mining operations are moving deeper into the earth, where coal seams exhibit increasingly pronounced characteristics of high ground stress, strong gas adsorption, and low permeability, such that a significant transformation occurs in the mining environment^3–7^. The mining environment has undergone significant changes, particularly during coal extraction. The buildup of stress within the coal rock mass comprising the coal seam and adjacent rocks can exceed the strength of these rocks, to suddenly release, which leads to the occurrence of geological hazards like rock bursts. These events pose a severe risk to the safety of underground mining operations^8–10^. Micro-seismic monitoring is a geophysical technique used for monitoring subterranean activities. This real-time geophysical monitoring technology utilizes micro-seismic signals emitted during the stress-induced fracturing of coal and rock to assess their stability. The seismic signals obtained by sensors within the mining area are used to analyze the timing, location, and energy source of micro-seismic events, for monitoring and early warning of potential geological hazards including rock bursts during coal mining^11,12^. In recent years, micro-seismic monitoring technology has been widely used in coal mines. For monitoring low-pressure conditions such as coal mine impact ground pressure and other mining-related disasters^13,14^.

Micro-seismic signals, as typical time-series data, evolve over time to reflect the dynamic properties of geological structures at specific moments^15^. In recent years, many work focused on time correlation analysis of micro-seismic signals by using deep learning algorithms. Lin et al.^16^ designed a self-similar convolutional neural network (SS-Net) to reduce the adverse effect of random noises. Zhu et al.^17^ introduced logistic regression into mine pressure hazard prediction in order to provide early warning signals of mine safety accidents. Batch gradient descent and Adagrad optimization algorithms were used in to enhance the model’s predictive performance and computational speed and accuracy. To enhance the prediction accuracy of micro-seismic events in rock burst prone mines, a MEA-BP neural network model was developed in^18^.

Micro-seismic signals, differing from typical industrial process signals, exhibit nonlinear and nonsmooth time series traits that complicate the accurate prediction of microseismic signals using a single model algorithm with significant short-term fluctuations. Similar approaches have been successfully applied in coal mine safety monitoring, where Chen et al.^19^. demonstrated the effectiveness of dual variational mode decomposition combined with LSTM for time series prediction, while Zhang et al.^20,21^. and Xu et al.^22^. showed that modal decomposition methods can significantly improve prediction accuracy for complex mining environment data.

In recent years, research efforts have been dedicated to analyzing nonlinear non-stationary signals through signal decomposition methods, such as Empirical Mode Decomposition (EMD). EMD retains the time-scale features of signals and can be integrated with time series prediction algorithms to improve prediction^23,24^. Zang et al.^25^ designed a long- and short-term memory network (LSTM) with residual attention mechanism for short-term prediction. Additionally, re-decomposing high-frequency intrinsic modal functions were used to improve the prediction accuracy of nonstationary time series data after empirical modal decomposition^26^. This approach has an advantage on extracting hidden information and potential temporal features from complex data. Wang et al.^27^. used the fuzzy entropy affiliation function as the threshold criterion of entropy to improve the adaptive ability of the model to the nonlinear and non-stationary data such that the prediction accuracy is improved.

To deal with the challenges posed by nonlinear and non-stationary characteristics of micro-seismic signals, this study employs a quadratic modal decomposition approach to process the data. Building upon the success of modal decomposition methods in similar applications, particularly the effectiveness of CEEMDAN in handling complex mining environment data^22^, this approach aims to extract more comprehensive temporal features from micro-seismic signals. The micro-seismic data is firstly decomposed into multiple Intrinsic Mode Functions (IMFs) by using the Complete Ensemble Empirical Mode Decomposition with Adaptive Noise (CEEMDAN). These IMFs are then clustered into high-frequency, low-frequency, and trend components by using fuzzy entropy. Subsequently, the high-frequency and low-frequency components are further decomposed by using the Variational Mode Decomposition (VMD) method. The obtained new IMFs are merged with historical micro-seismic signal data to form new multivariate time-series prediction data. A time-series prediction model integrating Temporal Convolutional Network (TCN) and Transformer is utilized to forecast the sequences. The model’s predictive performance is validated through analysis of micro-seismic monitoring data collected from an actual coal mine working face in Xinjiang.

## Materials and methods

### CEEMDAN

Empirical mode decomposition (EMD) is an adaptive decomposition method for analysing nonlinear and non-stationary time series data by decomposing a complex signal into a series of simple integrated mean modal functions (IMFs). The adaptive noise-complete ensemble empirical modal decomposition (CEEMDAN) is an improved algorithm of EMD, which removes the modal aliasing problem in EMD by adaptively adding Gaussian white noise to the original signal data; at the same time, the IMFs obtained by averaging the data with multiple additions of Gaussian white noise are more stable and accurate, and the mitigation of the boundary effects in EMD improves the stability and reproducibility of the decomposition. stability and repeatability of the decomposition^28^. Given the nonlinear and non-stationary nature of micro-seismic signals, this study employs CEEMDAN as the initial decomposition method. Compared to EMD, CEEMDAN effectively resolves modal aliasing issues; relative to EEMD, CEEMDAN utilizes an adaptive noise mechanism that automatically adjusts according to signal characteristics, thereby reducing computational complexity while maintaining decomposition quality; and compared to VMD, CEEMDAN requires no predefined parameters, rendering it more suitable for processing micro-seismic signals with complex time–frequency characteristics. Furthermore, CEEMDAN’s advantages in handling boundary effects and delivering reproducible results render it more robust within practical coal mine monitoring environments.

The specific CEEMDAN decomposition process for micro-seismic energy time series data is as follows:

1. First, Gaussian white noise with mean 0 is added to the time series data set ${\mathbf{Y}}(t)$ to construct $n$ sequences to be decomposed:
  1 $${\tilde{\mathbf{Y}}}(t) = {\mathbf{Y}}(t) + \varepsilon {{\varvec{\updelta}}}(t)$$
  where $\varepsilon$ is the weight coefficient matrix of Gaussian white noise;$\varepsilon {{\varvec{\updelta}}}(t)$ is the added Gaussian white noise.
2. The EMD decomposition of the added noise data ${\tilde{\mathbf{Y}}}(t)$ is performed to obtain the first order modal function ${\mathbf{IMF}}_{1}^{i} (t)$, the resulting $n$ modal functions are summed and averaged to obtain the first IMFs of the CEEMDAN decomposition ${\mathbf{IMF}}_{C1} (t)$, and the resulting ${\mathbf{IMF}}_{C1} (t)$ is subtracted from the original signal, i.e.:
  2 $${\mathbf{IMF}}_{C1} (t) = \frac{1}{n}\sum\limits_{i = 1}^{n} {\mathbf{IMF}}_{1}^{i} (t)$$
3. The remaining signal ${\mathbf{R}}_{{\mathbf{1}}} (t)$ is used as the new original signal, and so on repeating Eqs. (4) and (5) to extract the remaining CEEMDAN intrinsic modal components ${\mathbf{IMF}}_{Cn} (t)$ until the remaining signal ${\mathbf{R}}_{k} (t)$ cannot satisfy the decomposition condition. Finally, the original signal ${\mathbf{Y}}(t)$ is decomposed into a number of eigenmodal components ${\mathbf{IMF}}_{S}$ and a residual trend ${\mathbf{R}}_{k} (t)$, which is decomposed by the CEEMDAN algorithm with the expression:
  3 $${\mathbf{Y}}(t) = \sum\limits_{i = 1}^{k} {{\mathbf{IMF}}_{i} } + {\mathbf{R}}_{k} (t)$$

### Principles of micro-seismic signal time series prediction

The methods and principles of mine impact pressure monitoring are mainly divided into rock mechanics and geophysical methods, and the micro-seismic method belongs to one of the geophysical methods. The micro-seismic method mainly captures the electromagnetic radiation and vibration wave signals transmitted outward when the fissures are produced by the mining stress of the coal rock body, and realizes the continuous monitoring of the stress and deformation state of the coal rock body, so as to achieve the real-time early warning of the underground impact ground pressure. The energy intensity of the collected micro-seismic signals represents the degree of rupture of the coal rock body, and the continuous large intensity of the micro-seismic energy signals represents that the coal rock body is susceptible to secondary disasters caused by the impact ground pressure. At present, the mine mainly deploys multiple sets of sensors on the acquisition and monitoring surface to achieve real-time monitoring of vibration waves, and the acquired data contain information on the time, location and energy intensity of micro-seismic events. Therefore, the monitoring signals generated by micro-seismic activities can be recognized as a collection of micro-seismic time series data within a certain period of time.

There is a certain pattern of change in the intensity of micro-seismic energy over time, and there is a correlation between the energy of the micro-seismic event that occurs at the next time step and the energy of the micro-seismic event that occurs at the previous time step^29^. The energy of the micro-seismic event at the next time step is related to that of the previous time step. Assuming that the micro-seismic energy signals sampled by the micro-seismic monitoring system with a given time length of  $T$ are represented by the time-series data set ${\mathbf{Y}}(T)$:

4 $${\mathbf{Y}}(T) = \{ y(t_{1} ),y(t_{2} ),y(t_{3} ), \ldots ,y(t_{n} )\}$$

By dividing ${\mathbf{Y}}(T)$ by a time window of fixed size $\Delta t$, there exists a nonlinear mapping $f$ such that the time series data $y(t_{j} )$,$y(t_{j - 1} )$,$y(t_{j - 2} )$,…$y(t_{j - Dt} )$ within the time window is mapped to give the micro-seismic energy data $y(t_{j + 1} )$ for the next time step, denoted as:

5 $$y(t_{j + 1} ) = f(\{ y(t_{j} ),y(t_{j - 1} ),y(t_{j - 2} ),\ldots,y(t_{j - Dt} )\} )$$

**Fig. 1:**
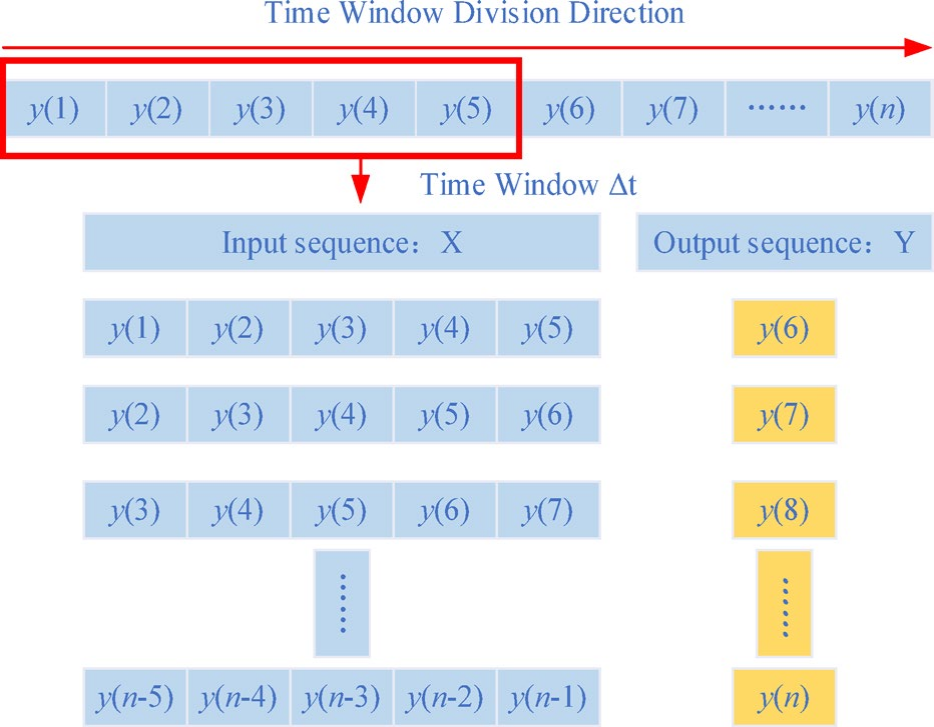
Segmentation of the time series data window.

Therefore, a time window can be used to divide the overall time series dataset ${\mathbf{Y}}(T)$, and the time series prediction of the future time step energy signal data can be achieved by constructing a neural network model. The specific time series data window division is shown in Fig. 1.

To avoid feature redundancy and ensure the validity of extracted features, this study employs fuzzy entropy to assess the complexity of IMFs obtained through CEEMDAN decomposition. Components are categorised into three types—high-frequency, trend, and low-frequency—based on their information content. The high-frequency and low-frequency components, possessing higher information content, undergo secondary VMD decomposition; the trend component, being relatively stable, is retained directly. This strategy aims to minimise ineffective expansion and information duplication.

### Fuzzy entropy

In signal processing and data analysis, entropy is used to measure the complexity and predictability of data. Fuzzy entropy is a method used to measure the complexity of a time series, which evaluates the complexity of the series by considering the similarity between points in the time series. Compared to sample entropy, which directly uses a hard threshold to determine similarity, fuzzy entropy defines the similarity between vectors by using a fuzzy function, which makes it more robust to noise. The fuzzy entropy can classify and reorganize the components more accurately after the micro-seismic data have been decomposed by CEEMDAN with the intrinsic modal function.

For the time series data set ${\mathbf{Y}}(t) = \{ y(t_{1} ),y(t_{2} ),...,y(t_{n} )\}$, the fuzzy entropy is used as follows:

1. Embedding the time series ${\mathbf{Y}}(t)$ in the dimension $m$ and initializing it, the resulting reconstructed sequence is:
  6 $${\hat{\mathbf{Y}}}(t) = \{ y(t_{1} ),y(t_{2} ),y(t_{3} ),...,...,y(t_{n + m - 1} )\} - {\mathbf{u}}(n)$$
  7 $${\mathbf{u}}(n) = \frac{1}{m}\sum\limits_{k = 0}^{m - 1} {{\mathbf{y}}(t_{n + k} )}$$
  where $${\mathbf{\overset{\lower0.5em\hbox{$\smash{\scriptscriptstyle\frown}$}}{Y} }}(t)$$ is the reconstructed sequence, $$n \in [1,n - m + 1]$$; $${\mathbf{u}}(n)$$ is the mean value of the $$m$$ continuous variable $${\mathbf{y}}(t_{n} )$$.
2. Calculate the distance between any two vectors ${\hat{\mathbf{Y}}}(t_{i} )$ and ${\hat{\mathbf{Y}}}(t_{j} )$ using the maximal paradigm $d_{ij}^{m}$ with the expression:
  8 $$d_{ij}^{m} = \max [{\hat{\mathbf{Y}}}(t_{i} ) - {\hat{\mathbf{Y}}}(t_{j} )]{\kern 1pt} {\kern 1pt} {\kern 1pt} {\kern 1pt} {\kern 1pt} {\kern 1pt} {\kern 1pt} {\kern 1pt} {\kern 1pt} {\kern 1pt} {\kern 1pt} {\kern 1pt} 1 \le i,j \le n - m + 1,i \ne j$$
3. Define a fuzzy affiliation function $\mu$ to measure the similarity between ${\hat{\mathbf{Y}}}(t_{i} )$ and ${\hat{\mathbf{Y}}}(t_{j} )$ , viz:
  9 $$\mu _{{ij}} = \left\{ {\begin{array}{*{20}l} {1,d_{{ij}}^{m} = 0} \hfill \\ {\exp \left[ { - \ln 2\left( {\frac{{d_{{ij}}^{m} }}{r}} \right)^{2} } \right],d_{{ij}}^{m} > 0} \hfill \\ \end{array} } \right.$$
  where $r$ is a constant parameter to adjust the sensitivity of the affiliation function to distance.
4. For the sequence ${\hat{\mathbf{Y}}}(t)$ the fuzzy similarity between each vector is calculated in turn, i.e.:
  10 $$\Phi^{m} (r) = \frac{1}{n - m + 1}\sum\limits_{i = 1}^{n - m} { \left(\frac{1}{n - m + 1}\sum\limits_{{{\mathbf{j = 1}}}}^{{{\mathbf{n - m + 1}}}} {u(d_{ij}^{m} )} \right)}$$
  where: $n$ is the parameter of the fuzzy function to adjust the steepness of the fuzzy function.
5. Increase the embedding dimension by 1, and repeat the above calculation of fuzzy similarity between vectors for $m + 1$ dimensional vectors to get $\Phi^{{{\mathbf{m + 1}}}} (r)$ , and obtain the fuzzy entropy and then complete the reclassification of the intrinsic components after CEEMDAN decomposition according to the proximity of the entropy value.
  11 $$FE(m,r,n){\mathbf{ = }}{\text{l}} {\text{n}} \left( {\frac{{\Phi^{m} (r)}}{{\Phi^{m + 1} (r)}}} \right)$$

The size of the fuzzy entropy value measures the complexity between the time series data, and the larger the fuzzy entropy value is, the more information the corresponding IMF component decomposed by CEEMDAN has. Therefore, the IMF components obtained by primary decomposition of time series data are determined by calculating the fuzzy entropy value of the components, reclassifying the IMF components of different sizes according to the entropy value, and secondary decomposition of IMF components with higher entropy value using the VMD variational modal decomposition, to further obtain the time series features in the sequence.

During the secondary decomposition process, the original signal and IMF components may exhibit differences in their characteristic scales. The ‘CEEMDAN → fuzzy entropy clustering → VMD’ strategy employed herein constrains each component within relatively stable frequency bands. The fixed-length sliding window further ensures consistency in the temporal scale, thereby mitigating the risk of cross-channel scale inconsistencies.

### VMD

Variational mode decomposition (VMD) is a completely non-recursive and adaptive signal decomposition method for non-stationary time series data^30^. VMD mainly decomposes a series of IMF components with different center frequencies from complex time series data, and obtains the optimal center frequencies by iterating the IMF components through the alternating direction multiplier algorithm, so as to facilitate further analysis and processing of the time series data.

The specific VMD decomposition core process focuses on constructing the variational model and solving the variational problem. The constrained model is shown in Eq. 13, and the detailed solution process is described in the literature^31^:

12 $$\left\{ \begin{gathered} \mathop {\min }\limits_{{\{ u_{k} ,\omega_{k} \} }} \left\{ {\sum\limits_{k = 1}^{K} {\left\| {\partial_{t} \left[ {\left( {(\delta (t) + \frac{j}{\pi t}} \right)u_{k} (t)} \right]e^{{ - j\omega_{k} t}} {\kern 1pt} } \right\|_{2}^{2} } } \right\} \hfill \\ {\text{s}} .t.\sum\limits_{k}^{K} {u_{k} = f.} \hfill \\ \end{gathered} \right.$$

### TCN network

**Fig. 2:**
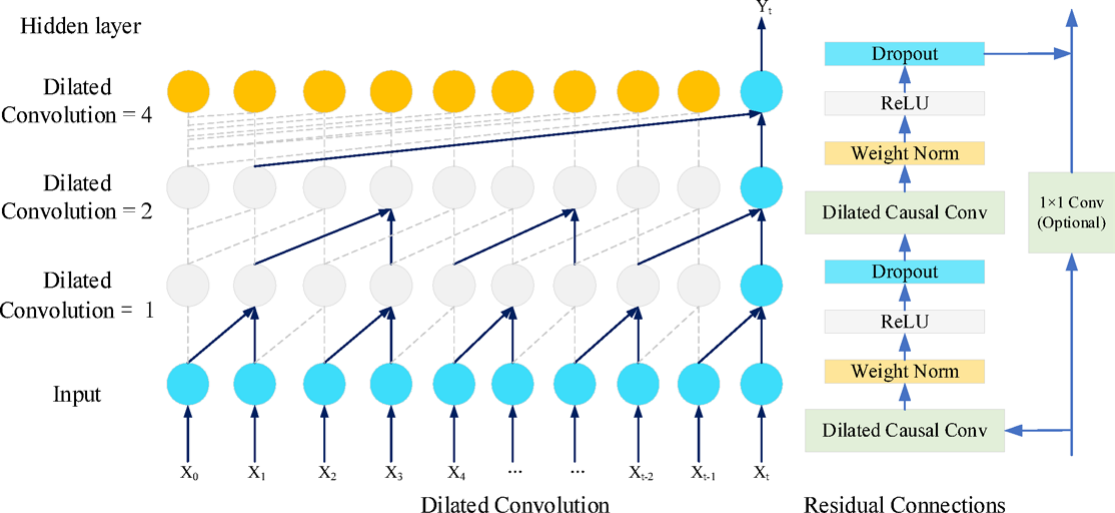
Structure of TCN network.

Temporal Convolution Network (TCN) is a neural network architecture specifically designed for processing time series data. Compared with the traditional convolutional neural network which only uses causal convolution for local feature extraction of time series data, TCN introduces residual connection and expansion convolution on top of the causal convolution structure. By adjusting the expansion coefficient of the expansion convolution to expand the receptive field, the temporal correlation can be captured from different receptive fields. Secondly, the residual connection added between the convolutional layers also avoids the possible gradient vanishing problem of CNN networks as much as possible, which effectively accelerates the training of model^32^. Taking the convolutional kernel size as 2 and the expansion factor as^1,2,4^ as an example, the TCN network model is shown in Fig. 2.

In our implementation, each residual block contains two layers of one-dimensional causal convolution, with a kernel size of k = 2 and an expansion factor of^1,2,4,8^. The effective receptive field R calculation is $R \approx 1 + 2\left( {k - 1} \right)\sum d = 1 + 2\left( {2 - 1} \right)\left( {1 + 2 + 4 + 8} \right) = 31$ steps, providing sufficient coverage for capturing long-term time dependencies.

### Transformer network

**Fig. 3:**
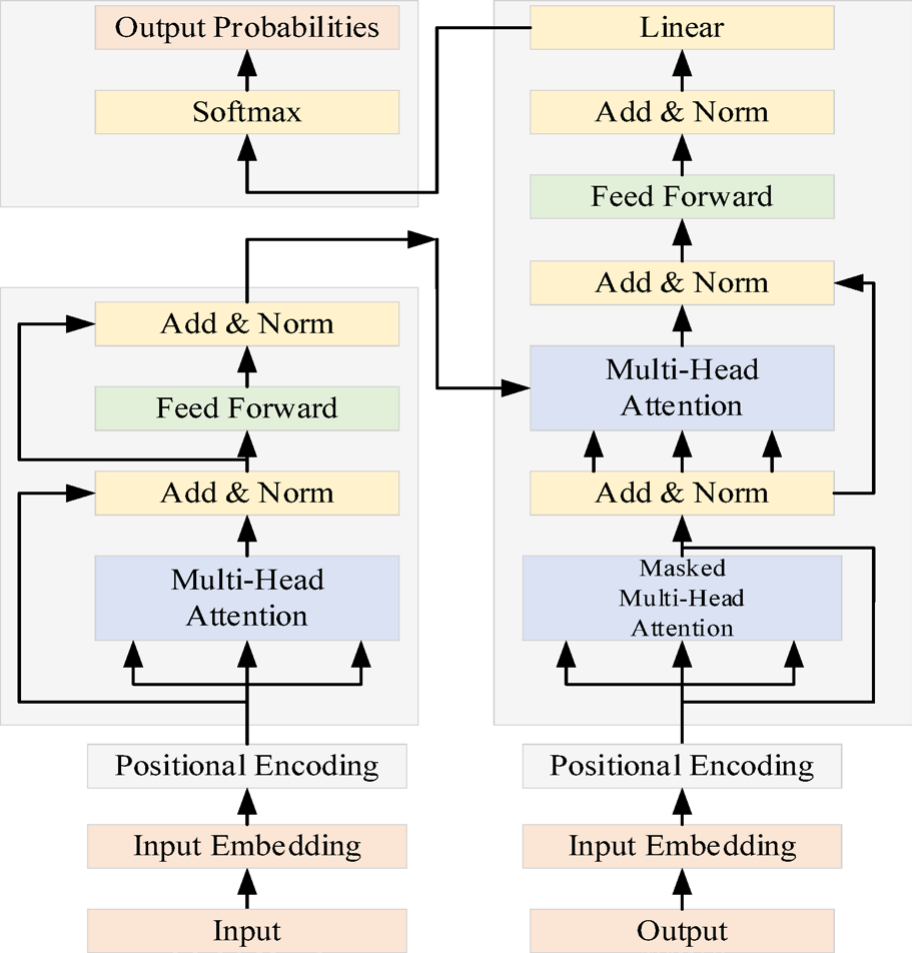
Transformer network structure diagram.

Transformer is an end-to-end model suitable for solving time series. Different from traditional recurrent neural networks, Transformer relies on the self-attention mechanism to capture the information of different locations in space, which effectively solves the problem of the dependence of traditional recurrent neural networks and their transformer networks on medium- and long-term time prediction. As shown in Fig. 3, the Transformer model is mainly composed of two parts, encoder and decoder, which can be stacked together with multiple encoders and decoders of the same number, and contains a multi-head attention mechanism consisting of multiple self-attention mechanisms running in parallel.

Transformer’s self-attention mechanism can be formulated as follows:

13 $${\mathbf{A(Q,K,V)}} = \mathbf{S}\left(\frac{\mathbf{Q}\mathbf{K}^T}{\sqrt{d_K}}\right)\mathbf{V}$$

where ${\mathbf{A}}$ represents the attention mechanism;${\mathbf{S}}$ represents the SoftMax function that calculates the attention weights;${\mathbf{Q}},{\mathbf{K}},{\mathbf{V}}$ calculates the matrix of the attention mechanism, the keys of the matrix and the values, respectively; $d_{K}$ is the dimension of the keys.

The multi-attention mechanism can be formulated as follows:

14 $${\mathbf{M}}_{h} = {\mathbf{C}}({\mathbf{h}}_{1} ,{\mathbf{h}}_{2} ,{\mathbf{h}}_{3} ,\ldots,{\mathbf{h}}_{i} ){\mathbf{W}}_{o}$$

15 $${\mathbf{h}}_{i} = {\mathbf{A}}({\mathbf{QW}}_{Qi} ,{\mathbf{QW}}_{Ki} ,{\mathbf{QW}}_{Vi} )$$

where ${\mathbf{M}}_{h}$ is the multi-attention mechanism; ${\mathbf{C}}$ is the connection mechanism between the attention; ${\mathbf{h}}_{i}$ denotes the $i$ attention mechanism; ${\mathbf{W}}_{o}$ is the linear transformation weight matrix after the connection of the multi-attention mechanism; ${\mathbf{W}}_{Qi} ,{\mathbf{W}}_{Ki} ,{\mathbf{W}}_{Vi}$ is the linear transformation weight matrix.

For micro-seismic signals exhibiting non-stationary and non-linear characteristics, this study employs standard absolute position coding. The quadratic decomposition method effectively addresses non-stationarity by mapping slow-varying and non-stationary components to low-frequency and trend channels respectively. A fixed sliding window strategy ensures that segments entering the attention layer are approximately stationary within the window, thereby reducing sensitivity to specific position encoding variants. The TCN component captures local ordered dependencies, while the trend channel provides long-term background information. This enables standard absolute position encoding to sufficiently meet task requirements while maintaining implementation simplicity and reproducibility.

For unavoidable residual weak coupling, the TCN and Transformer components at the model end undergo adaptive alignment during end-to-end training through the local constraints of convolutions, the global weighting of attention mechanisms, and LayerNorm normalization. This further mitigates their detrimental impact on prediction performance.

It is worth noting that although the attention mechanism of the Transformer can effectively capture the dependencies among temporal features, its attention weights mainly reflect the intensity of the model’s use of context information rather than a direct mapping of specific physical mechanisms. In practical applications, the physical interpretation of attention weights should be treated with caution to avoid directly equating them with the specific frequency band characteristics of rock fractures.

## Model

### Data decomposition

Based on the principle of micro-seismic signal time series prediction, it is evident that the process involves segmenting the input features and output labels of micro-seismic signals according to the time window size, and utilizing these segments as proportionally divided training, testing, and validation sets for the deep learning algorithm. Empirical Mode Decomposition (EMD) is a method for analyzing non-smooth and non-linear signals through the decomposition into a series of Intrinsic Mode Functions (IMFs).While EMD performs well in many aspects, it faces limitations, particularly with signals that have nested frequencies or similar-scale frequency components. Extensive research indicates that, in contrast to the feature sequences derived from primary decomposition, those from secondary decomposition more effectively capture the signal’s internal characteristics, leading to enhanced time series prediction accuracy. Consequently, this study employs a quadratic modal decomposition approach to process micro-seismic signals, extract multiple IMFs, and integrate these with the raw micro-seismic signals to construct a multi-input single-output dataset, applying a fixed time window to segment the dataset, thereby fulfilling the training requirements of deep learning models.

**Fig. 4:**
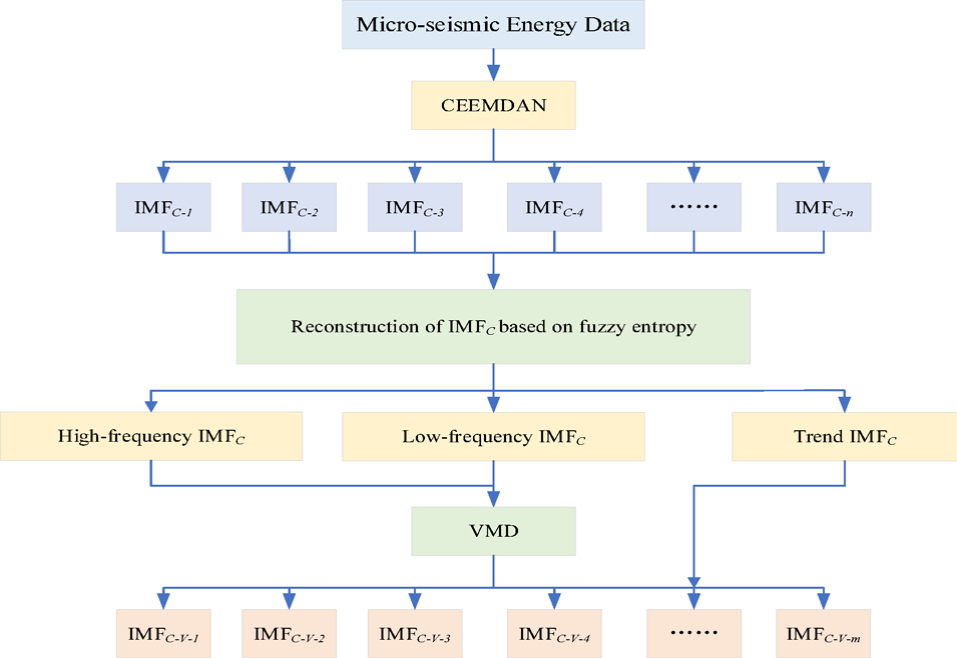
Flow chart of secondary decomposition of micro-seismic signal.

The overall secondary decomposition process of the micro-seismic signal is shown in Fig. 4. Firstly, the micro-seismic signal data are subjected to a primary empirical modal decomposition using CEEMDAN, and the obtained $n$ IMFs and residual components after decomposition are denoted as ${\mathbf{IMF}}_{C}$. Then, the obtained ${\mathbf{IMF}}_{C}$ are evaluated for signal complexity and irregularity using fuzzy entropy, and based on the results of fuzzy entropy value calculation, ${\mathbf{IMF}}_{C}$ can be further classified into high frequency, low frequency and trend sequences. Among them, ${\mathbf{IMF}}_{C}$, which contains high frequency components, usually has high fuzzy entropy value, reflecting the fast changes and high dynamic characteristics of the signal; ${\mathbf{IMF}}_{C}$, which contains low frequency components, has low fuzzy entropy value, reflecting the slow changes and stability of the signal; and the final trend sequence, which represents the baseline trend of the signal, usually contains the information of the lowest frequency. Finally, the distinguished high-frequency ${\mathbf{IMF}}_{C}$ and low-frequency ${\mathbf{IMF}}_{C}$ components are fed into the VMD for quadratic decomposition, and the trend sequence is directly merged into the obtained $m$ newly generated IMFs, denoted as ${\mathbf{IMF}}_{C - V}$.

**Fig. 5:**
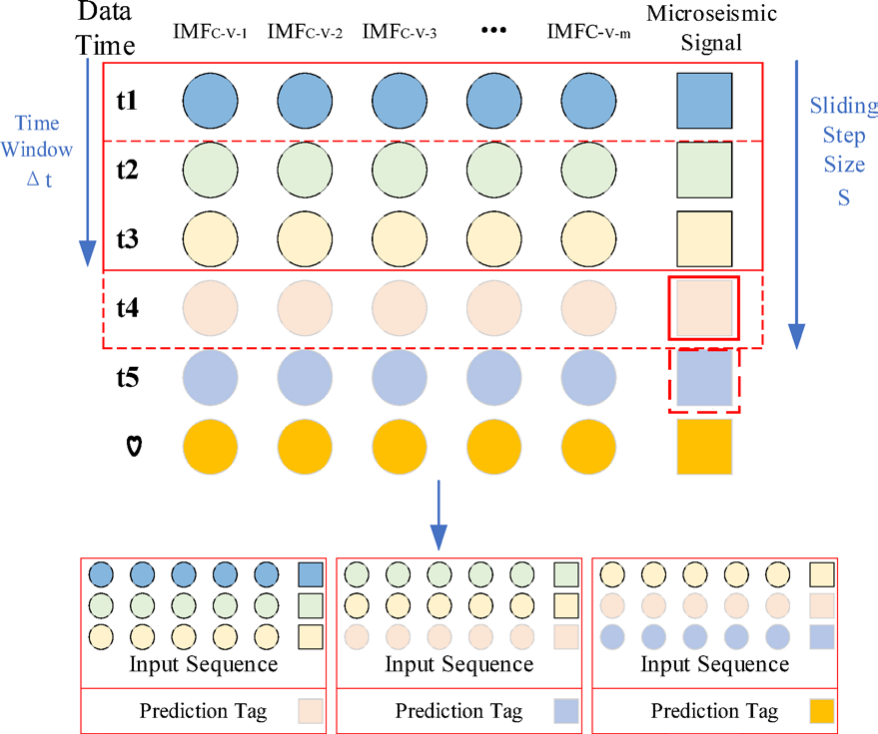
Flow chart of micro-seismic signal data construction.

The ${\mathbf{IMF}}_{C - V}$ component obtained after quadratic decomposition is combined with the original micro-seismic signal data to become a time series prediction dataset. The sampling sliding window method divides the training set by setting a window with a fixed size of $\Delta t$ and a sliding step size of $S$. The time series data within the obtained fixed window is continuous, where the same color indicates the temporal correlation between the features, and the delineated ${\mathbf{IMF}}_{C - V}$ component is used as the input feature data for the time series prediction, and the next time step micro-seismic signal data is used as the output label data. input feature data for time series prediction, and the micro-seismic signal data of the next time step is used as the output labelled data. The dataset construction process is shown in Fig. 5.

In VMD secondary decomposition, the selection of the number of modes K follows three principles: determining the quantity of primary frequency components within the signal by analyzing the spectral characteristics of reconstructed modes, employing center frequency matching to evaluate decomposition quality and prevent mode aliasing, and utilizing residual entropy values to assess decomposition completeness to ensure no information loss.

Spectral analysis of the reconstructed components reveals that primary energy concentrates within two frequency bands (15–25 Hz and 35–45 Hz), indicating the presence of two dominant frequency components. When K = 1, the center frequency of the decomposed mode highly aligns with the original signal frequency with a matching error below 3%, while when K = 2, the two modes correspond to the two distinct frequency components identified in spectral analysis with a center frequency interval exceeding 10 Hz to avoid mode overlap. The residual entropy values for K = 1 and K = 2 were 0.12 and 0.15 respectively, both maintained at acceptably low levels.

The decomposition process successfully captured core signal information without significant loss. Considering the practical characteristics of micro-seismic signals, this study selected K = 1 and K = 2 as decomposition parameters, ensuring adequate decomposition while avoiding computational redundancy caused by over-decomposition.

### Time series prediction module

**Fig. 6:**
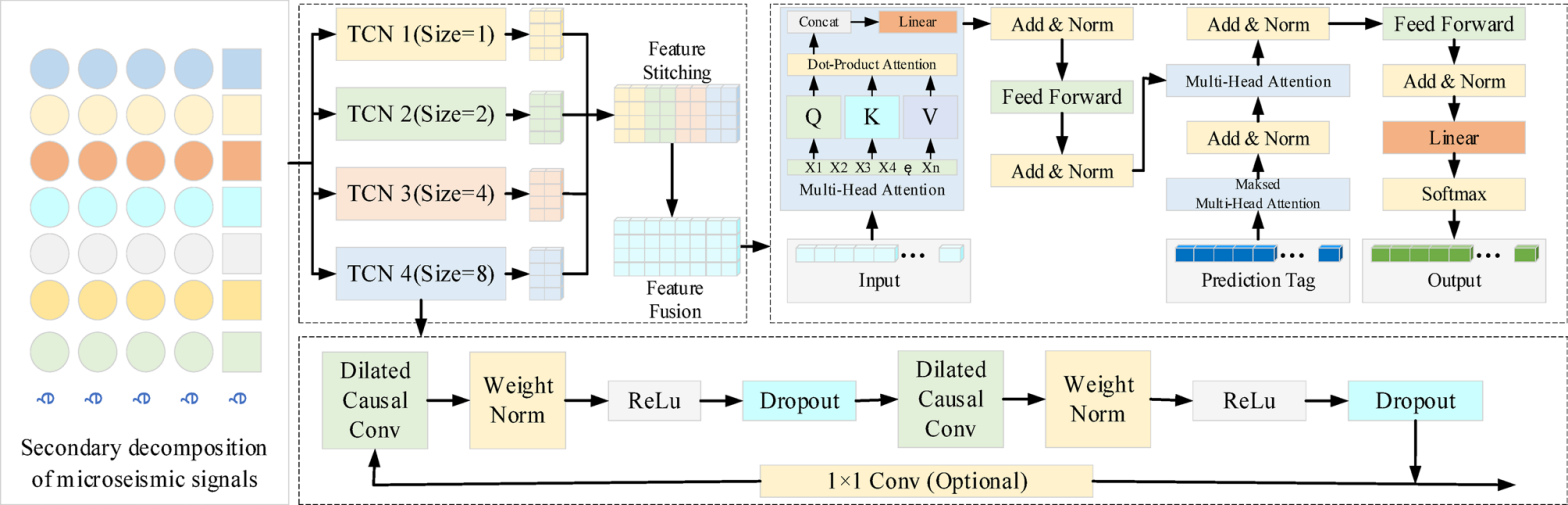
Network timing prediction process of TCN-Transformer.

Based on the previously constructed multivariate IMFs of micro-seismic signals and their input–output dataset, this study employs the TCN-Transformer algorithm as the predictive model for micro-seismic signals. The model comprises two main components: TCN and Transformer. The TCN is responsible for multi-scale feature extraction from the input time series data, The Transformer network, leveraging the attention mechanism, performs parallel computations on these features to accomplish time series prediction. The TCN model incorporates expansion convolution and residual linkage, with expansion convolution capable of adjusting receptive fields based on its expansion coefficient, allowing for the extraction of time series features across various scales. In contrast to traditional multi-scale CNNs, the TCN network directly propagates gradients through residual connections, unaffected by intermediate layers. This approach mitigates the issue of gradient vanishing, enabling the model to achieve a more profound representation of temporal features. The Transformer component receives the feature matrix extracted by TCN, and employs positional encoding to incorporate sequential information into the time series, followed by the application of multi-head attention mechanisms post-encoding. Once encoding is complete, the input features undergo processing through the multi-head attention mechanism, which concurrently considers the features at each time point along with their interrelationships, thereby enabling the model to learn from diverse representational subspaces. Concurrently, the Transformer decoder employs a masking attention mechanism to prevent the leakage of future information, ensuring that predictions for the current time point are based solely on information available up to that point. Additionally, the decoder incorporates an additional attention layer to focus on the encoder’s output. The final output of the decoder is presented in Fig. 6.

The components derived through secondary decomposition exhibit relatively stable scale characteristics within fixed sliding windows. The integration of TCN’s local feature extraction with Transformer’s global attention mechanism achieves effective fusion of multi-scale features. LayerNorm and attention weighting mechanisms dynamically adjust the contribution of each component during training, avoiding the complexity of manual weighting fusion while preserving the model’s end-to-end trainability.

## Experiment

### Experimental setup and dataset

The micro-seismic signal originated from a coal mine in Urumqi, Xinjiang. The production conditions of this mine are complex, and the lithology of the coal-bearing strata of its mine is mainly claystone, mudstone, muddy siltstone and siltstone, which are plastic rocks; the structure of the rock body is a thin bedded structure, and the top and bottom plates of the coal beds are mostly claystone, mudstone, and silty sandy mudstone, with local pseudo-topping of charcoal mudstone, which are all low-mechanical-strength rocks.

The ARAMIS M/E micro-seismic monitoring system has been installed in the underground working face of the mine, with a strike length of 728.84 m and a mineable length of 519.82 m. To validate the effectiveness and accuracy of the model presented in this paper for predicting micro-seismic signals, micro-seismic monitoring data from the working face for the year 2022 has been selected for the experiment.

The selection of baseline models is based on three key factors: application scenario compatibility, computational resource constraints, and real-time deployment requirements. Micro-seismic signal prediction falls within the realm of short-sequence, high-frequency real-time industrial monitoring scenarios, necessitating low-latency response capabilities. While large-scale models such as Informer and Autoformer demonstrate exceptional performance in large-scale, long-sequence prediction tasks, they present risks of overfitting in short sequences, excessive computational resource demands, and insufficient real-time capabilities, rendering them unsuitable for micro-seismic signal prediction applications. This study selected LSTM, CNN-LSTM, CNN-LSTM-Attention, and Transformer as baseline models. These models feature moderate parameter scales and reasonable computational complexity, are widely applied in micro-seismic signal prediction, and are representative of the field.

All baseline models (LSTM, CNN-LSTM, CNN-LSTM-Attention, Transformer) were trained and selected under identical data partitioning and training protocols as the proposed model. Consistent data preprocessing, fixed sliding windows, and training/validation/test split ratios were employed. Baseline models underwent lightweight grid/range searches on their respective key structural hyperparameters (e.g., learning rate, hidden dimensions/layers, attention heads, dropout, etc.). Optimal configurations were selected based on validation metrics (prioritising MAE while considering RMSE), with results reported on the test set to ensure fair comparison.

**Fig. 7:**
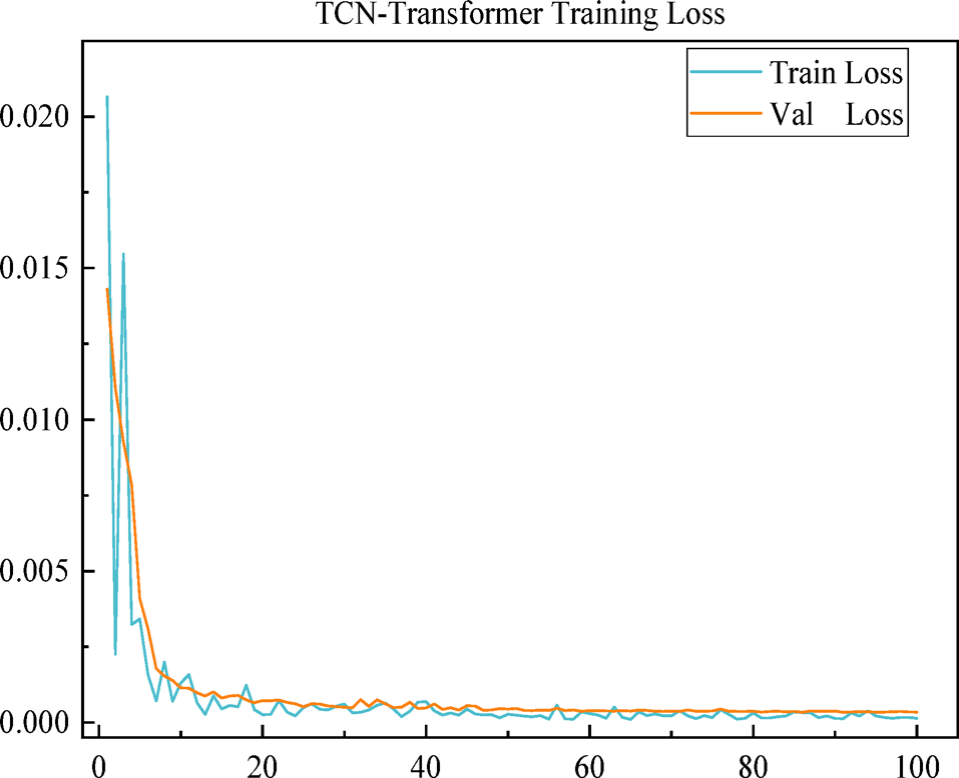
Training loss curve of TCN-Transformer model.

All experiments were performed on an AMD Ryzen 7 6800H CPU with a 3.2 GHz clock speed, an NVIDIA GeForce GTX 3060 GPU with 6 GB of RAM, 16 GB of system RAM, Python 3.9.15, and Pytorch 1.13.1 with CUDA 11.6. The key parameters for the TCN and Transformer models encompass the TCN’s input dimensions, network layers, expansion coefficient, output dimensions, and the Transformer’s embedding dimensions, hidden layer size, number of attention mechanism heads, encoder-decoder stacks, and training iterations, among others. Specific hyperparameters are detailed in Table 1. An early stopping strategy was employed during experiments to prevent overfitting and enhance training efficiency. The optimal model was subsequently evaluated using the validation set, with the overall training loss depicted in Fig. 7.

**Table 1:** TCN-Transformer parameters.

Parameters	TCN-transformer
TCN Input Dimension	15
TCN Network Layers	4
Dilation Convolution Factor	1,2,4,8
TCN Output Dimension	15
Dimension	64
Transformer Hidden Layer Size	64
Heads of Attention Mechanisms	8
Number of Encoder-Decoder Stacks	2
Max/Min Learning Rate	1e-3/5e-5
Epoch	100

### Evaluation metrics

This paper assesses the prediction accuracy of the TCN-Transformer model using the goodness of fit $R^{2}$, mean square error $MSE$, mean absolute error $MAE$ and mean absolute percentage error $MAPE$ as evaluation metrics. These metrics indicate model performance, and the lower the value or closer to 1, the better the accuracy. The calculation formulas for these metrics are as follows:

16 $$R^{2} = 1 - \frac{{\sum\limits_{i = 1}^{n} {(\tilde{y}_{i} - y_{i} )^{2} } }}{{\sum\limits_{i = 1}^{n} {(\tilde{y}_{i} - y)^{2} } }}$$

17 $$MSE = \frac{1}{n}\sum\limits_{i = 1}^{n} {\left( {\hat{y}_{i} - y_{i} } \right)^{2} }$$

18 $$MAE = \frac{1}{n}\sum\limits_{i = 1}^{n} {\left| {\hat{y}_{i} - y_{i} } \right|}$$

19 $$MAPE = \frac{1}{n}\sum\limits_{i = 1}^{n} {\left| {\frac{{\hat{y}_{i} - y_{i} }}{{y_{i} }}} \right|} \times 100\%$$

where $\tilde{y}_{i}$ is the value of blind denoising process; $y_{i}$ is the value of pure data; $\hat{y}_{i}$ is the value of time series prediction.

### Secondary empirical decomposition of micro-seismic signals

**Fig. 8:**
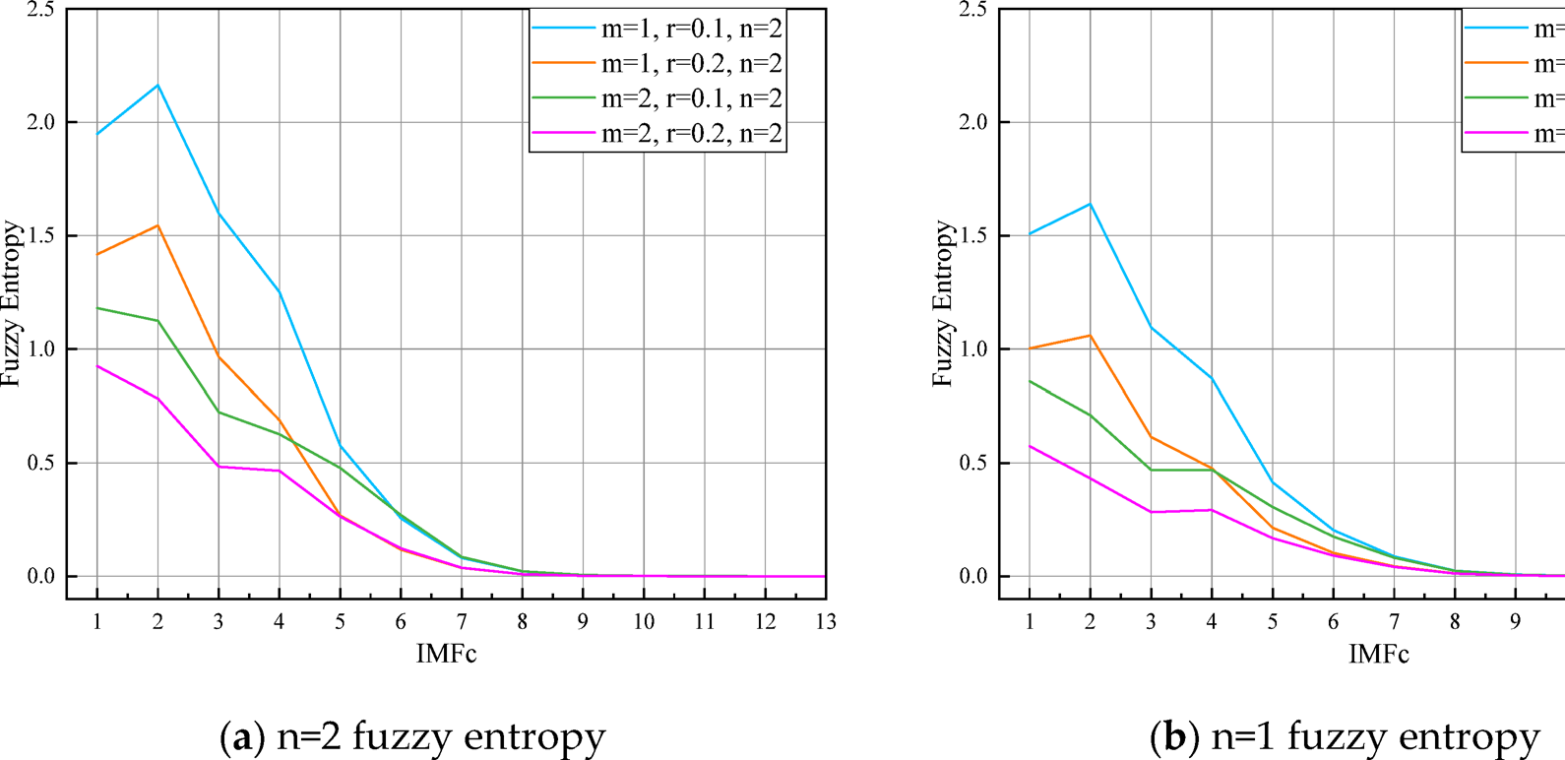
Fuzzy entropy of IMF-c after a decomposition.

The micro-seismic signal undergoes initial decomposition via the Complete Ensemble Empirical Mode Decomposition with Adaptive Noise (CEEMDAN) technique. The resultant 13 intrinsic modal functions (IMFs), represented as ${\mathbf{IMF}}_{C}$, are presented in Fig. 8. For each ${\mathbf{IMF}}_{C}$, the fuzzy entropy is computed to assess the complexity of the information within the time series data. Key parameters for calculating fuzzy entropy include the embedding dimension $m$, similarity threshold $r$, and parameters $n$ of the fuzzy function. Fuzzy entropy values for the ${\mathbf{IMF}}_{C}$ components, calculated under varying parameters, are displayed in Fig. 8.

Observing the figure, one can note that the fuzzy entropy values for the ${\mathbf{IMF}}_{C}$ components decrease sequentially, aligning with the frequency characteristics revealed by the CEEMDAN decomposition. This pattern indicates a progressive reduction in the complexity of the feature information encapsulated within the time series data.

Qin, et al.^33^ and Wang et al.^34^. have demonstrated that calculating the sample entropy for the IMFs resulting from CEEMDAN decomposition, and subsequently recombining similar simple modes based on their entropy complexity, can enhance computational efficiency and accuracy while mitigating the risk of overfitting.

Consequently, this paper employs fuzzy entropy calculations on the ${\mathbf{IMF}}_{C}$ components derived from CEEMDAN-decomposed micro-seismic signals. The calculated entropy values categorize the ${\mathbf{IMF}}_{C}$ into high-frequency, trending, and low-frequency components.

**Fig. 9:**
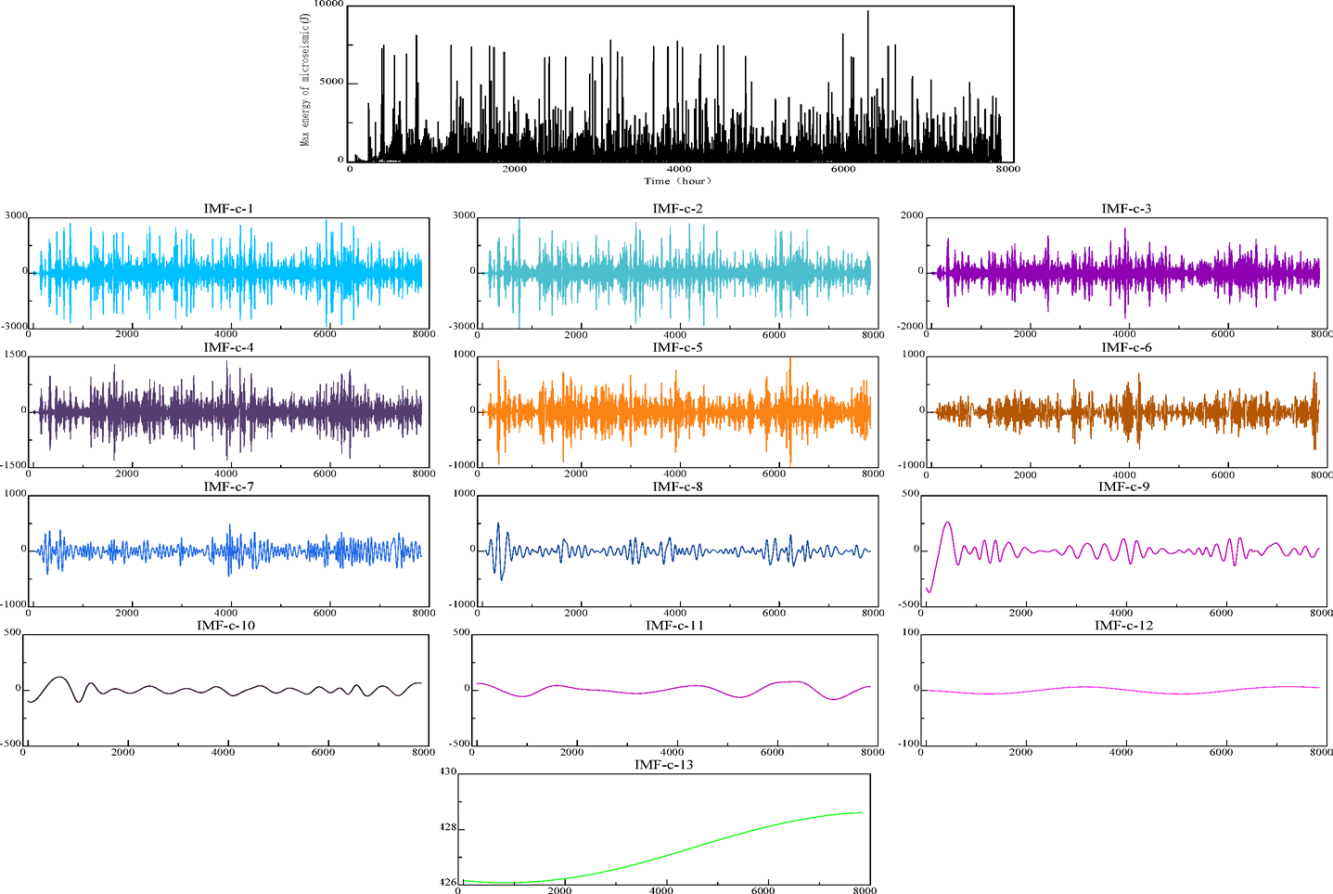
CEEMDAN decomposition results.

**Fig. 10:**
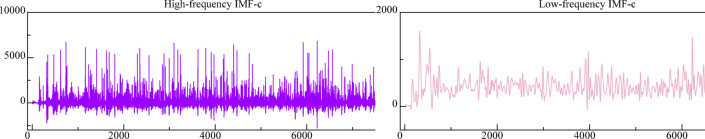
Reconstruction components of high and low frequency IMF-c.

As illustrated in Fig. 9 by the fuzzy entropy values, the first four components, ${\mathbf{IMF}}_{C - 1}$ through ${\mathbf{IMF}}_{C - 4}$, exhibit high fuzzy entropy. This indicates that these ${\mathbf{IMF}}$ components are characterized by frequent and irregular fluctuations and the time series undergoes rapid changes within these components, a hallmark of the time series’ nonlinear dynamics. The fuzzy entropy values for ${\mathbf{IMF}}_{C - 5}$ and ${\mathbf{IMF}}_{C - 6}$ are lower than those of the high-frequency components. However, they are higher than those of the subsequent ${\mathbf{IMF}}$ components. In time series analysis, components with moderate fuzzy entropy values often indicate the periodicity and trends within the series, thus they are identified as the trend components. Fuzzy entropy values that are lower, particularly from ${\mathbf{IMF}}_{C - 7}$ to ${\mathbf{IMF}}_{C - 13}$, tend to expose the fundamental trends and the most regular patterns within the time series data. As such, these are categorized as low-frequency ${\mathbf{IMF}}$ components. The high-frequency and low-frequency ${\mathbf{IMF}}_{C}$ components, once identified, are individually reconstructed through a cumulative process. The outcomes of these reconstructions are visually presented in Fig. 10.

**Fig. 11:**
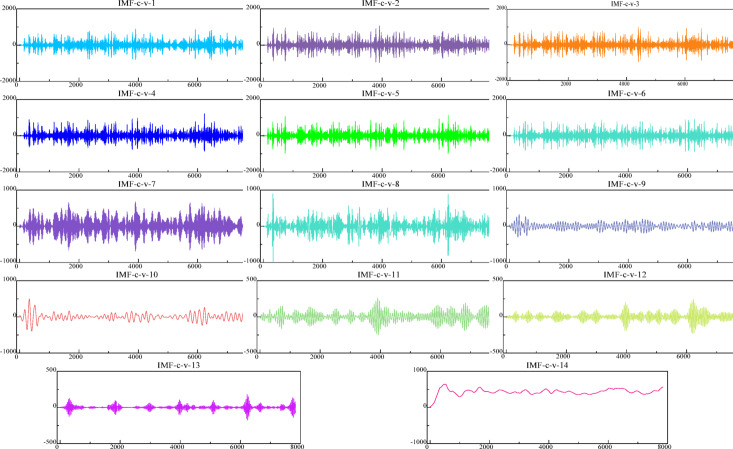
VMD secondary decomposition results of high and low frequency components.

Ultimately, the reconstructed components undergo a two-stage decomposition process utilizing Variational Mode Decomposition (VMD). The resultant ${\mathbf{IMF}}_{C - V}$ component, derived from this secondary decomposition, is combined with the trend ${\mathbf{IMF}}$ component to create a comprehensive input sequence. This sequence is then fed into the subsequent time series prediction network. The outcomes of the VMD’s secondary decomposition are graphically represented in Fig. 11.

### Time series prediction of micro-seismic signals

Following the quadratic decomposition, the ${\mathbf{IMF}}_{C - V}$ component, extracted from the micro-seismic signal source data, serves as the dataset for multi-featured time series prediction. A sliding time window method is employed to segment the dataset into time series windows. These windows are then allocated to the training, validation, and test sets at a ratio of 65%, 15%, and 20%, respectively, to facilitate model training.

**Fig. 12:**
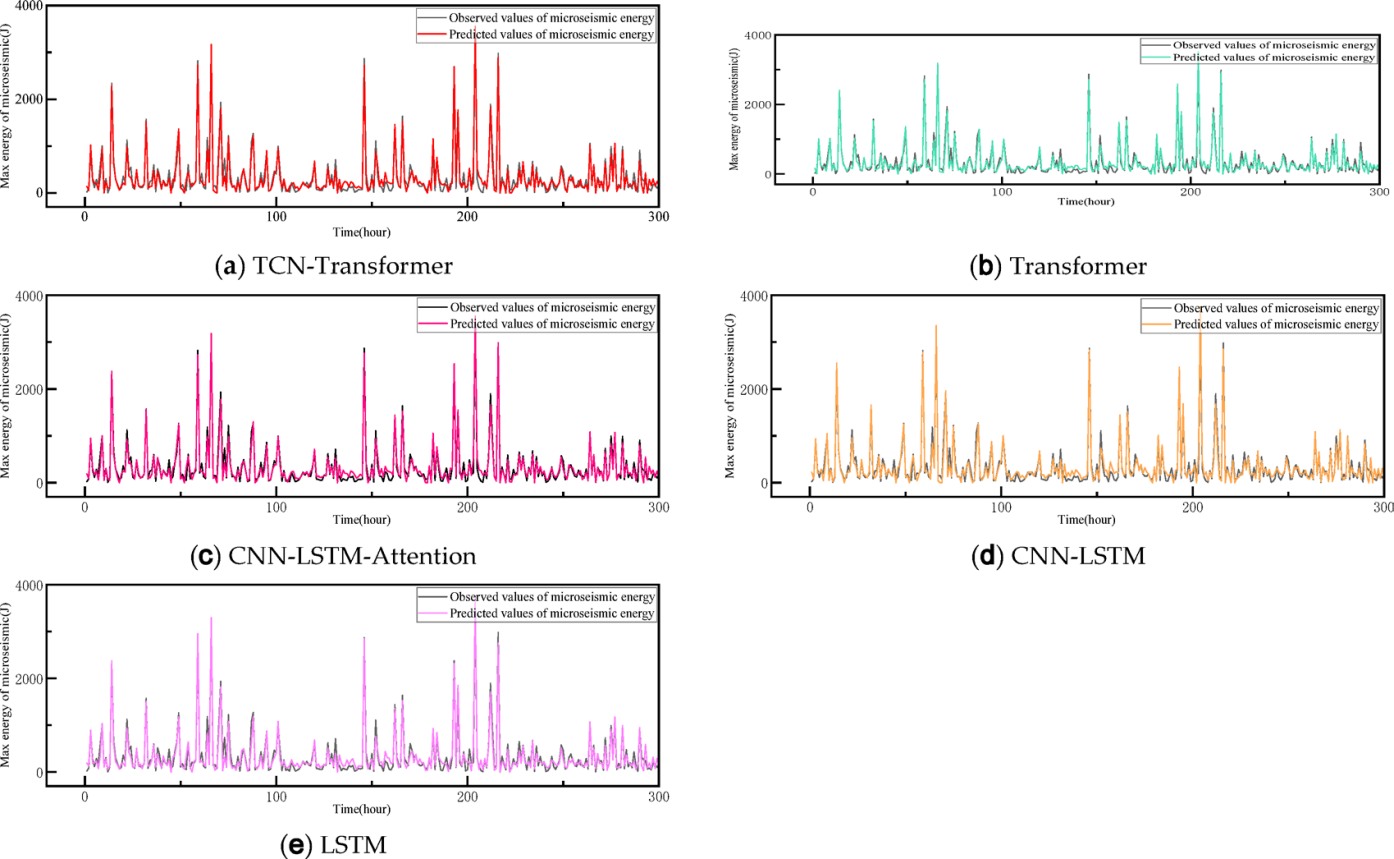
Prediction results of maximum energy of micro-seismic signals by each method.

**Fig. 13:**
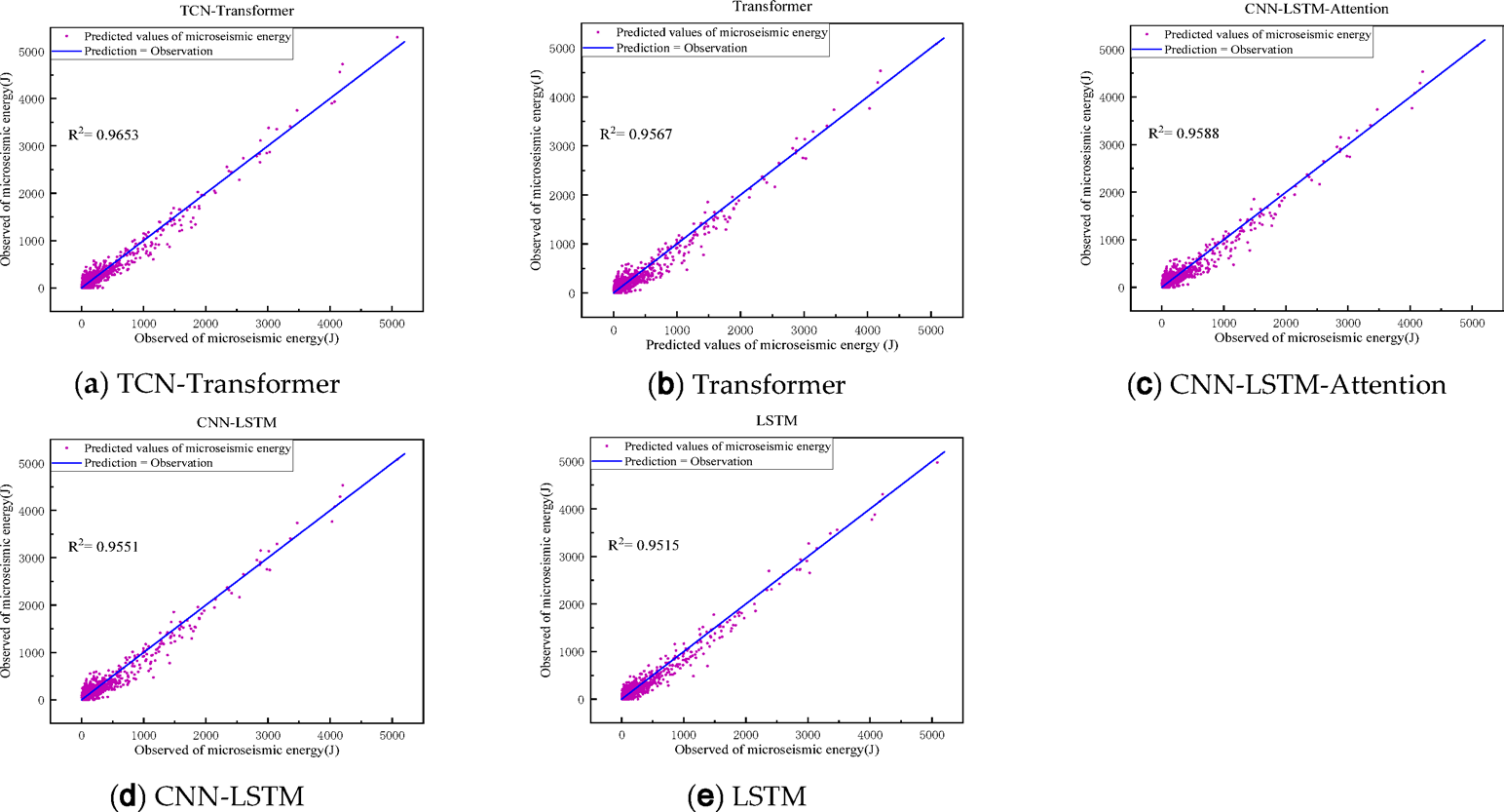
Prediction results of maximum energy of micro-seismic signals by each method.

To further assess the performance of the models presented in this paper, we introduce and train several models using the same dataset: the Transformer ablation model, an enhanced LSTM based on recurrent neural networks, a multiscale convolutional long short-term memory network (CNN-LSTM), and a CNN-LSTM incorporating an attention mechanism. The outcomes of these models are depicted in Figs. 12 and 13, while the average predictive performance is detailed in Table 2.

**Table 2:** Comparison of prediction accuracy of different algorithms.

Methodologies	R^2^	RMSE	MAE	MAPE
LSTM	0.952	135.8	99.4	0.883
CNN-LSTM	0.955	130.6	96.5	0.850
CNN-LSTM-Attention	0.959	125.1	93.9	0.845
Transformer	0.957	128.3	96.2	0.849
TCN-Transformer	**0.965**	**114.7**	**84.7**	**0.835**

The LSTM model generally maintains stable prediction results, thanks to its ability to capture temporal data characteristics through state retention. However, enhancements to recurrent neural network-based models can introduce latency, potentially degrading predictive performance. The enhanced CNN-LSTM model, equipped with a multi-scale convolutional neural network, efficiently extracts temporal features across various scales, particularly the CNN-LSTM-Attention model, which benefits significantly from the inclusion of an attention mechanism. This allows it to effectively process both global and local features, thereby rendering the prediction outcomes of both the CNN-LSTM-Attention and the attention-based Transformer models more precise.

The TCN-Transformer model advantages stem from the TCN’s capacity to capture feature information across various scales via its diverse receptive fields, enabling the TCN-Transformer to process comprehensive feature information akin to the CNN-LSTM-Attention model. It also effectively leverages local feature information within the data for learning purposes. Furthermore, compared to traditional CNNs, the TCN’s residual connections and dilated convolutions not only capture the causal relationships within temporal data, but also reduce the model’s parameter count, thereby enhancing predictive accuracy and maintaining a lightweight network architecture, making it particularly well-suited for real-time data prediction in practical applications.

## Ablation experiment

To assess the effectiveness of the quadratic decomposition, fuzzy entropy, and TCN-Transformer models introduced in this paper, this section examines: (1) the prediction of maximum energy in micro-seismic time series data without decomposition, (2) the prediction of maximum energy in micro-seismic time series data using initial decomposition methods, (3) a comparison of various quadratic decomposition techniques, employing fuzzy entropy(FE) and sample entropy(SE) as criteria for predicting maximum energy in micro-seismic time series.

### Time series forecasting without empirical modal decomposition

**Fig. 14:**
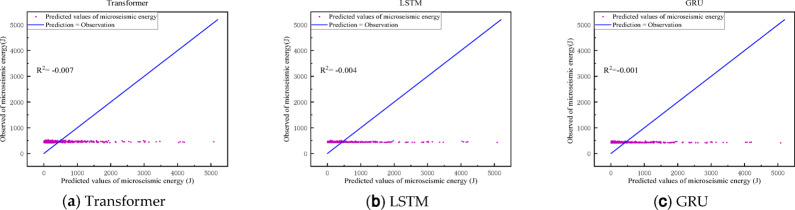
Prediction results of maximum energy of micro-seismic signals by each method.

In this section, we predict the original micro-seismic signal’s maximum energy values using GRU, LSTM, and Transformer models, which are key players in the deep learning time series prediction domain. The dataset is split into training, testing, and validation sets at the original scale for input into these various models. The predictions made by each model are detailed in Table 3, with corresponding plots of predicted versus actual values presented in Fig. 14. Utilizing the original one-dimensional signals directly in deep learning prediction methods, these models struggle to capture temporal features, and the predictions fail to exhibit temporal correlation with actual values. This suggests that micro-seismic energy signals, being inherently nonlinear and nonsmooth, pose challenges to directly derive temporal correlations. Thus, employing empirical modal decomposition or similar techniques to extract intrinsic temporal features is essential for ensuring accurate predictions.

**Table 3:** Comparison of prediction accuracy of different algorithms.

Methodologies	R^2^	RMSE	MAE	MAPE
LSTM	− 0.004	618.1	397.6	4.053
GRU	− 0.001	617.2	389.9	3.910
Transformer	− 0.007	619.2	403.8	4.177

### Time series forecasting with empirical modal decomposition

**Fig. 15:**
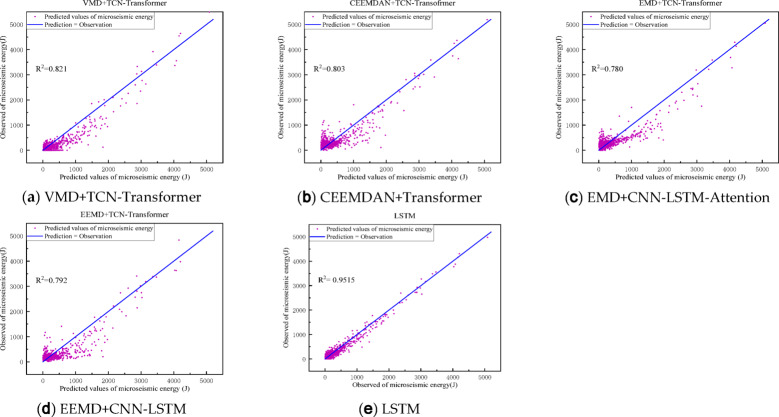
Prediction and fitting diagram of the maximum energy of a decomposition micro-seismic signal.

In this section, we aim to assess the impact of various primary empirical modal decomposition techniques on the final prediction outcomes, we apply the commonly used EMD, EEMD, VMD, and CEEMDAN methods to decompose the signals individually, and then organize the decomposed data into training, testing, and validation sets as described in the previous section, Subsequently, these sets are input into the TCN-Transformer model for prediction. The predictions from each model are presented in Table 4, with corresponding plots of predicted versus actual values in Fig. 15. The findings indicate that VMD-decomposed data yields the most effective predictions, Other methods perform slightly less effectively than VMD, highlighting that micro-seismic energy signals without feature modal decomposition pose challenges for time series prediction models to discern intrinsic temporal features, whereas empirical modal decomposition prior to modeling enables the model to more precisely capture these temporal characteristics, thereby facilitating the micro-seismic energy signal time series prediction task.

**Table 4:** Comparison of prediction accuracy of different algorithms.

Methodologies	R^2^	RMSE	MAE	MAPE
EMD	0.780	289.2	185.3	1.411
EEMD	0.792	280.9	196.1	1.861
VMD	**0.821**	**265.6**	**183.4**	**0.798**
CEEMDAN	0.803	273.2	189.8	1.880

### Time series forecasting under quadratic decomposition

The purpose of this section is to evaluate the efficacy of fuzzy entropy and sample entropy in component reconstruction following initial empirical mode decomposition, as well as their role in predicting micro-seismic energy signal time series when applied to secondary decomposition of the reconstructed results. This assessment serves to validate the theoretical advantages of the CEEMDAN selection discussed in Section “CEEMDAN”. This process begins with the initial decomposition of the raw signal through EMD, EEMD, VMD, and CEEMDAN, followed by the reconstruction of these decomposed components using fuzzy entropy and sample entropy. Subsequently, a secondary decomposition is conducted on these reconstructed results using EMD, EEMD, VMD, and CEEMDAN in sequence. The components derived from this final decomposition are analyzed for consistency with the initial method, and the resultant dataset is then input into the TCN-Transformer model for predictive analysis. The outcomes of these experiments are presented in Tables 5, 6, 7, 8, demonstrating a marked enhancement in prediction accuracy for the time series post-secondary decomposition as opposed to predictions made after primary decomposition alone.

**Table 5:** Prediction results of EMD and different quadratic decomposition algorithms.

Methodologies	R^2^	RMSE	MAE	MAPE
EMD + SE + EMD	0.790	279.3	175.2	1.51
EMD + SE + EEMD	0.802	274.1	170.1	1.47
EMD + SE + VMD	0.883	210.6	145.4	1.23
EMD + SE + CEEMDAN	0.871	220.5	150.3	1.42
EMD + FE + EMD	0.783	285.0	180.2	1.57
EMD + FE + EEMD	0.795	278.7	173.4	1.54
EMD + FE + VMD	**0.892**	**202.0**	**141.5**	**1.10**
EMD + FE + CEEMDAN	0.865	215.3	148.5	1.37

**Table 6:** Prediction results of EEMD and different quadratic decomposition algorithms.

Methodologies	R^2^	RMSE	MAE	MAPE
EEMD + SE + EMD	0.815	273.1	173.6	1.33
EEMD + SE + EEMD	0.805	276.8	176.2	1.36
EEMD + SE + VMD	0.850	250.3	160.1	1.11
EEMD + SE + CEEMDAN	0.831	260.7	170.4	1.21
EEMD + FE + EMD	0.801	279.5	179.8	1.39
EEMD + FE + EEMD	0.815	273.0	173.0	1.33
EEMD + FE + VMD	**0.855**	**248.7**	**158.4**	**1.07**
EEMD + FE + CEEMDAN	0.835	258.0	168.5	1.17

**Table 7:** Prediction results of VMD and different quadratic decomposition algorithms.

Methodologies	R^2^	RMSE	MAE	MAPE
VMD + SE + EMD	0.865	202.3	142.1	1.12
VMD + SE + EEMD	0.875	192.7	132.4	1.07
VMD + SE + VMD	0.925	150.6	100.3	0.91
VMD + SE + CEEMDAN	0.948	131.8	91.2	0.89
VMD + FE + EMD	0.870	198.6	137.8	1.10
VMD + FE + EEMD	0.880	188.9	127.6	1.04
VMD + FE + VMD	0.930	145.2	95.4	0.89
VMD + FE + CEEMDAN	**0.953**	**126.7**	**90.5**	**0.87**

**Table 8:** Prediction results of CEEMDAN and different quadratic decomposition algorithms.

Methodologies	R^2^	RMSE	MAE	MAPE
CEEMDAN + SE + EMD	0.871	198.6	137.8	1.10
CEEMDAN + SE + EEMD	0.880	188.9	127.6	1.04
CEEMDAN + SE + VMD	0.952	125.0	90.1	0.87
CEEMDAN + SE + CEEMDAN	0.950	130.7	92.4	0.88
CEEMDAN + FE + EMD	0.873	195.6	135.1	1.08
CEEMDAN + FE + EEMD	0.885	185.3	122.4	1.03
CEEMDAN + FE + VMD	**0.965**	**114.7**	**84.7**	**0.83**
CEEMDAN + FE + CEEMDAN	0.947	135.2	94.9	0.91

It is important to note that micro-seismic energy signals are often sampled amidst some noise interference. The use of fuzzy entropy in the primary decomposition components is more effective at handling noise interference in real-world downhole conditions than sample entropy, leading to enhanced prediction accuracy and overall model robustness. Among empirical modal decomposition methods, EMD is straightforward but noise-sensitive, potentially resulting in unstable decomposition and less refined predictive outcomes. EEMD addresses EMD’s limitations by incorporating noise auxiliary signals, significantly enhancing final prediction results. VMD and CEEMDAN demonstrate greater robustness and stability against noise interference, with prediction accuracy notably surpassing that of EMD and EEMD. VMD excels at managing nonlinear and nonsmooth signals through variational mode decomposition, albeit with increased computational complexity. CEEMDAN integrates the benefits of EEMD and adaptive noise, further bolstering decomposition stability and accuracy, and achieves the highest prediction accuracy and robustness, particularly when combined with VMD.

## Conclusions and future work

With the rapid development of information technology, micro-seismic monitoring and early warning systems are facing new opportunities and challenges. Based on the quadratic mode decomposition and TCN-Transformer framework proposed in this paper, its application potential in multi-source data fusion can be further explored. By combining micro-seismic signals with geophysical data such as electromagnetic radiation, acoustic emission, and ground deformation, a more comprehensive rock behaviour analysis system can be constructed. This fusion can not only make up for the limitations of a single data source, but also improve the reliability of the early warning system through the complementary information between different sensors. The main contributions of this paper are reflected in the following three aspects:

1. CEEMDAN was used for the initial decomposition of micro-seismic signals. The IMFs were classified and reconstructed by using fuzzy entropy, and subsequently decomposed secondary with VMD. Simulation results indicated that secondary modal decomposition more effectively uncovers latent features within micro-seismic signals, substantially enhancing time series prediction accuracy.
2. The TCN network captured multi-scale temporal features via dilated convolution and residual connections. At the same time, the Transformer network parallelized time series data processing through its self-attention mechanism. This combined approach effectively captured both local and global features within time series data, thereby enhancing model prediction accuracy.
3. Compared to traditional time series prediction models, the quadratic modal decomposition and TCN-Transformer-based framework presented here exhibited superior fitting and prediction accuracy with micro-seismic signals. From verification with micro-seismic monitoring data from an active coal mine in Xinjiang, the presented model showed the capacity to accurately forecast future micro-seismic signal states for enhancing early warning systems for geological hazards, including impact ground pressure.

Based on the results and limitations of the current study, future work can be carried out in the following aspects. Existing fusion algorithms are still insufficient in dealing with the heterogeneity of different data sources, and there is a need to develop smarter fusion strategies that can dynamically adjust the weights of each data source according to data quality and relevance. Meanwhile, the adaptive ability of the model also needs to be improved, as there are obvious differences in the geological conditions and states of different mining environments, and neural network architectures that can adapt to these changes need to be developed. Additionally, future research should focus on systematic early warning threshold optimization strategies and ROC/PR curve analysis based on specific mining areas’ actual requirements, as well as conducting attention mechanism and physical mechanism correlation experiments to obtain qualitative/quantitative consistency evidence. These future research directions will not only help to advance micro-seismic monitoring and early warning technology, but will also make an important contribution to the safety and efficiency of mining operations.

## Data Availability

The datasets generated during and/or analyzed during the current study are available from the corresponding author on reasonable request.
